# Effect of Immunophilin Inhibitors on Cochlear Fibroblasts and Spiral Ganglion Cells

**DOI:** 10.1159/000526454

**Published:** 2022-09-08

**Authors:** Madeleine Goblet, Thomas Lenarz, Gerrit Paasche

**Affiliations:** ^a^Department of Otorhinolaryngology, Hannover Medical School, Hannover, Germany; ^b^Hearing4all Cluster of Excellence, Hannover Medical School, Hannover, Germany

**Keywords:** Immunophilin inhibitor, Spiral ganglion neuron, Inner ear, Cell survival, Inner ear therapy

## Abstract

**Introduction:**

Loss of hair cells and degeneration of spiral ganglion neurons (SGN) lead to severe hearing loss or deafness. The successful use of a cochlear implant (CI) depends among other factors on the number of surviving SGN. Postoperative formation of fibrous tissue around the electrode array causes an increase in electrical impedances at the stimulating contacts. The use of immunophilin inhibitors may reduce the inflammatory processes without suppressing the immune response. Here, we report on in vitro experiments with different concentrations of immunophilin inhibitors MM284 and compound V20 regarding a possible application of these substances in the inner ear.

**Methods:**

Standard cell lines (NIH/3T3 fibroblasts), freshly isolated SGN, and fibroblasts from neonatal rat cochleae (p3–5) were incubated with different concentrations of immunophilin inhibitors for 48 h. Metabolic activity of fibroblasts was investigated by MTT assay and cell survival by counting of immunochemically stained neurons and compared to controls.

**Results:**

MM284 did not affect SGN numbers and neurite growth at concentrations of 4 × 10<sup>−5</sup> mol/L and below, whereas V20 had no effect at 8 × 10<sup>−6</sup> mol/L and below. Metabolic activity of fibroblasts was unchanged at these concentrations.

**Conclusion:**

Especially MM284 might be considered as a possible candidate for application within the cochlea.

## Introduction

Immunophilins are a class of substances comprising among other cyclophilins (Cyps) and FK506-binding proteins (FKBP) [Barik, 2006]. Cyps play an important role in antiviral activity, cell regeneration, and inflammation and signal transduction [Wang et al., 2011; Flisiak and Parfieniuk-Kowerda, 2012]. The most abundant member of this family is the cytosolic cyclophilin A (CypA), which is representing 0.4% of total cellular proteins and can be found in human body at a concentration of 1 μg/mg [Daum et al., 2009; Flisiak and Parfieniuk-Kowerda, 2012]. Intracellular CypA is involved in cell signaling, calcium homoeostasis, and transport mechanism, whereas it is secreted in the extracellular space by neurons [Fauré et al., 2006], inflammatory cells, and upon cell death [Heinzmann et al., 2015]. Extracellular CypA shows proinflammatory cytokine-like behavior, is a potent chemoattractant for leukocytes, and elicits inflammatory responses [Pasetto et al., 2017]. Cyps are also cytosolic receptors for the immunosuppressive drug cyclosporine A (CsA) [Edlich et al., 2006]. CsA is a small peptide, inactive in acute inflammation but possesses a strong immunosuppressive action. Cyclophilins mediate the action of CsA by forming drug-dependent complexes [Liu et al., 1991]. The exclusive binding of CsA to the active site of CypA is the reason for many physiological effects such as mediation of immunosuppression, inhibition of the protein phosphatase activity of calcineurin, and thereby prevention of the cytokine gene transcription regulation [Edlich et al., 2006; Daum et al., 2009]. Human Cyp-CsA complexes prevent the transcription of genes involved in T-cell activation. This correlates with the specific block of the cellular immune response. CsA is clinically used as a potent immunosuppressant in the prevention of allograft rejection [Hacker and Fischer, 1993].

Cyp-CsA and FKBP-FK506 complexes bind to calcineurin [Barik, 2006]. Activation of calcineurin contributes to noise-induced hearing loss [Minami et al., 2004]. Application of CsA and FK506 was shown to decrease the threshold shift after acoustic injury [Uemaetomari et al., 2005]. At least for FK506 this effect is a combination of inhibition of calcineurin and an additional reduction in formation of reactive oxygen species [He et al., 2021]. Furthermore, FKBP12 is abundant throughout the cochlea and the dorsal cochlear nucleus [Zajic et al., 2001]. Cycloheximide (CHX) is an additional potent inhibitor of FKBP12, which additionally has neuroregenerative properties [Christner et al., 1999].

One nonimmunosuppressive CsA derivate is the immunophilin inhibitor MM284. This compound can only interact with cyclophilins extracellularly. Therefore, it reduces the recruitment of T cells and macrophages and leads to a reduction in inflammatory processes without affecting the immune system [Hacker and Fischer, 1993; Heinzmann et al., 2015].

A second immunophilin inhibitor is V20, a conjugate of CHX and CsA, linked via 2,2′-(ethylene dioxy)-diethylamin. V20 represents an active substance, which in complex with Cyps reduces the calcineurin inhibition and is therefore potentially nonimmunosuppressive. Due to its composition, this substance could potentially inhibit cyclophilins and FKBPs.

Sensory neural hearing loss is accompanied by loss of spiral ganglion neurons (SGN). In order to ensure the best possible care for these patients, remaining SGN can be electrically stimulated by a cochlear implant (CI). For this, a large number of vital SGN and a close nerve-electrode contact are necessary. A close nerve-electrode contact results in lower thresholds [Telmesani and Said, 2015] and less spread of excitation [Yang et al., 2020]. In addition, after implantation of the electrode into the cochlea, connective tissue forms around the electrode array, which impairs signal transmission to the nerve cells of the auditory nerve [Somdas et al., 2007]. Inflammation is one factor in formation of fibrous tissue [Velnar et al., 2009; Fernández-Klett and Priller, 2014]. Among current approaches for a reduction of fibrous tissue growth after cochlear implantation are microstructured surfaces [Reich et al., 2008], use of metal ions [Paasche et al., 2011], or application of dexamethasone by mini-osmotic pumps [Vivero et al., 2008] or by elution from the electrode [Wilk et al., 2016]. Despite all efforts, only application of steroids during electrode insertion [Prenzler et al., 2020] and incorporation of dexamethasone in the silicone body of the electrode array [Briggs et al., 2020] are used clinically. The two nonimmunosuppressive immunophilin inhibitors MM284 and V20 might also provide a means to reduce inflammatory processes after cochlear implantation. This could prevent formation of connective tissue around the CI electrode and provide an improved nerve-electrode contact. Therefore, the aim of the current study was to investigate in vitro possible toxic effects of both substances on SGN and fibroblasts and therefore to evaluate their safety for an intracochlear application in conjunction with cochlear implantation.

## Materials and Methods

### Statement of Ethics

The experiments were conducted in accordance with the German “Law on Protecting Animals” (§4) and the European Directive 2010/63/EU for protection of animals used for experimental purpose and registered (no. 2016/118) with the local authorities (Lower Saxony State Office for Consumer Protection and Food Safety [LAVES], Oldenburg, Germany). Sprague-Dawley rats of different sexes (postnatal days 3–5) were used for the experiments. All rats had free access to water and food and were kept at 22 ± 2°C under 14 h/10 h light/dark cycle.

### Preparation of Substances

Both immunophilin inhibitors, MM284 and V20 (G. Fischer, MPIBC, Halle/S., Germany), were dissolved in DMSO (#A3672; AppliChem GmbH, Darmstadt, Germany) at a concentration of 10 mM to generate a stock solution. Before addition to the cells (50 μL/well), each immunophilin inhibitor was first transferred in cell-specific culture medium (compare sections on cell culture) and diluted further to concentrations twice as high as the intended final concentrations of 2 × 10^−4^ mol/L to 2.56 × 10^−9^ mol/L per well. For SGN, this solution was additionally supplemented with 100 ng/mL brain-derived neurotrophic factor (BDNF; Invitrogen, Carlsbad, USA) (final concentration per well: 50 ng/mL).

### Fibroblast Cell Culture

Two different types of fibroblasts were used for the in vitro experiments: standard cell line NIH/3T3 (ATCC number CRL 1658) and primary fibroblasts from the rat cochlea. To prepare the latter, spiral ganglion cells were freshly prepared and isolated from neonatal Sprague-Dawley rats (postnatal days 3–5) as described earlier [Wefstaedt et al., 2005]. Starting from this protocol, fibroblasts were purified according to [Anacker et al., 2022]. Briefly, cells were cultivated in 25 cm^2^ cell culture flasks (TPP, Trasadingen, Switzerland) at a density of 3 × 10^5^ cells in 5 mL fibroblast medium (89% Dulbecco's Modified Eagle's Medium [DMEM #FG0445, high glucose; Biochrom, Berlin, Germany] with phenol red supplemented with 10% fetal calf serum [FCS; Biochrom] and 1% penicillin/streptomycin [Biochrom]). For purification, the cells were subcultivated at 80% confluence up to passage 3 (P3). To separate fibroblasts and glia cells, fluorescence-based flow cytometric cell sorting was used. The cells were sorted according to the fluorescence signal via antibodies specific for the cell types. For fibroblasts anti-Thy-1-antibody, clone OX-7, and FITC conjugated (MAB1406; Merck Millipore, Darmstadt, Germany) (ICC: 1/25) and for glia cells NGF Receptor p75 Antibody (192-IgG)-PE conjugated (Sc-71691; Santa Cruz Biotechnology Inc., Heidelberg, Germany) (ICC: 1/50) were used. For the spiral ganglion, neurons and fibroblasts were shown to be Thy1 positive [Fields et al., 1978; Raff et al., 1979], and Schwann cells, satellite cells, and neurons express p75NGFR [Pannese and Procacci, 2002; Whitlon et al., 2010]. The collected fibroblasts were centrifuged and the pellet was resuspended in 5 mL warm fibroblast medium and subcultivated at 37°C, 5% CO_2_ and 95% humidity for up to 10 passages.

For the experiments, NIH/3T3 and primary fibroblasts were subcultivated at 80% confluence (passage 3–5) and seeded in 96-multiwell culture plates (TPP) at a density of 8 × 10^4^ cells/well in 50 μL supplemented fibroblast medium. The outermost 36 wells of a plate were filled with Ca^2+^/Mg^2+^-free Hank's balanced salt solution with 0.35 g/L NaHCO_3_ but without phenol red (HBSS; Biochrom). Wells B2-G2 and B11-G11 contained complemented DMEM (blank) and untreated cells as control. The residual wells (B3-G10) were used for the substances or control series with the same concentrations of the solvent DMSO. The substances or DMSO solution were added at the time of plating (NIH/3T3) or after 24 h (primary cells). After this time, the medium was removed before adding 50 μL of fresh medium and 50 μL diluted substances or DMSO solution. To control wells 50 μL of fibroblast medium was added. Each experiment was repeated 6 times with *n* = 3 per plate (only primary fibroblasts and V20: *N* = 4). Cells were incubated for 48 h.

### MTT Assay

To measure metabolic cell activity, the MTT (3-[4,5-dimethylthiazol-2-yl]-2,5-diphenyltetrazoliumbromid) test was performed according to ISO 10993-5:2009 Appendix C (DIN EN ISO 10993-5:2009-10). At the day of the test, a solution of 1 mg/mL MTT (AppliChem) in DMEM without phenol red was prepared, sterile filtered through a PES membrane, pore size 0.22 mm (#SLGP033RS; EMD Millipore, Billerica, MA, USA), and stored until use at room temperature under exclusion of light. After incubation of the cells in the diluted substances, the supernatant was removed and 50 μL of the MTT solution was added per well (final concentration: 50 μg MTT/well). The cells were incubated for 2 h at 37°C, 5% CO_2_. After decanting the MTT solution, addition of 100 μL isopropanol (Sigma-Aldrich, St. Louis, MO, USA) per well dissolved the formazan crystals completely while shaking. Absorption was measured at 570 nm using a Synergy H1 Hybrid Reader (BioTek, Bad Friedrichshall, Germany). For each plate, the blank values were averaged and the mean value was subtracted from the measured values of all other wells. The results for control wells of untreated cells were also averaged and taken as 100% cell activity. Values for each tested concentration were averaged and normalized to the untreated controls of the same 96-well plate before results from different plates were averaged.

### Spiral Ganglion Cell Culture

The primary SGN were isolated from postnatal Sprague-Dawley rats (postnatal days 3–5) of different sexes. Dissection of the cochleae and dissociation of the spiral ganglia were performed according to the previously described protocol [Wefstaedt et al., 2005]. The separated spiral ganglia were enzymatically (HBSS with 0.1% trypsin [Biochrom] and 0.01% DNase I [Roche, Basel, Switzerland]) dissociated. Dissociation was stopped adding FCS. The cells were then triturated in 1 mL serum-free medium (Panserin 401 [PAN Biotech, Aidenbach, Germany], supplemented with HEPES buffer [23.4 μmol/mL; Invitrogen], phosphate-buffered saline [PBS; PBS tablets; Gibco® Thermo Fisher Scientific, Waltham, MA, USA], glucose [0.15%; Braun AG, Melsungen, Germany], penicillin [30 U/mL; Biochrom], N2 supplement [0.1%; Invitrogen], and insulin [8.7 μg/mL; Biochrom]) until a homogeneous solution was achieved. All viable cells of the dissociated spiral ganglia were counted in a Neubauer counting chamber (Brand GmbH, Wertheim, Germany) using trypan blue exclusion test (Sigma-Aldrich).

Finally, the cells were seeded at a density of 1 × 10^4^ cells/well in a 96-multiwell culture plate, coated with poly D/L-ornithine (0.1 mg/mL; Sigma-Aldrich) and laminin (0.01 mg/mL; natural from mouse; Life Technologies, Carlsbad, CA, USA). The SGN were cultivated for 48 h in a mixture of complemented Panserin, supplemented with BDNF (final concentration: 50 ng/mL), and the different dilutions (final concentrations: 2 × 10^−4^ down to 2.56 × 10^−9^ mol/L) of immunophilin inhibitors V20 and MM284 or control series with the same concentrations of the solvent DMSO. For each concentration and positive control (SGN with complemented Panserin supplemented with 50 ng/mL BDNF), three wells per plate were treated and every setup was repeated six times. After 48 h, the cells were fixed with a 1 + 1 mixture of acetone (J. T. Baker, Deventer, The Netherlands) and methanol (Carl Roth, Karlsruhe, Germany) for 10 min and washed 3 times with PBS.

### Immunohistochemistry

After 48 h incubation, the SGN were immuncytochemically stained. For primary staining, the cells were incubated with the monoclonal mouse 200 kDa neurofilament antibody (#NCL-L-NF200-N52, Novocastra; Leica, Wetzlar, Germany) for 1 h at 37°C, 5% CO_2_. After rinsing with PBS, the secondary biotinylated anti-mouse antibody (#VC-PK-6102; Vector Laboratories Inc., Burlingame, CA, USA) was added for 30 min at room temperature. After washing again with PBS, ABC complex solution (#VC-PK-6102, Vectastain® Elite® ABC-Kit; Vector Laboratories) was added to the cells using the protocol of the Vectastain® Elite® ABC Kit. Addition of diaminobenzidine solution (#SK-4100, Peroxidase Substrate Kit DAB; Vector Laboratories) visualized the stained SGN.

### Data Evaluation (SGN)

Surviving neurons were defined as neurofilament-positive cells exhibiting a neurite length of at least three cell soma diameters [Gillespie et al., 2001]. All surviving neurons of each well were manually counted using a transmission light microscope (Olympus CKX41, Hamburg, Germany) with a camera (Colorview III, SIS; Olympus). For neurite length measurements, the five longest neurons in each field of view (one in the center and four around the perimeter of the well) were manually traced by using the imaging software cellSens (Olympus) [Schmidt et al., 2018]. The survival rate was calculated by the number of surviving neurons with reference to BDNF-treated controls (mean number of neurons in the positive control) of the same plate and then averaged across different plates (*N* = 6). The same procedure was followed for evaluation of neurite length.

### Statistical Analysis

Statistical analysis was performed using GraphPad Prism version 5.02 (GraphPad, La Jolla, CA, USA). As tests for Gaussian distribution are not very meaningful for small N, nonparametric tests were used. To account for matched observations, Friedman test followed by Dunn's posttest was used to compare results with different concentrations of a treatment group to untreated controls. To compare different treatment groups at a specific concentration, Kruskal-Wallis's test followed by Dunn's posttest was applied. *p* values of less than 0.05 were considered to be statistically significant.

## Results

### Effects of Immunophilin Inhibitors on Fibroblasts

**Fig. 1:**
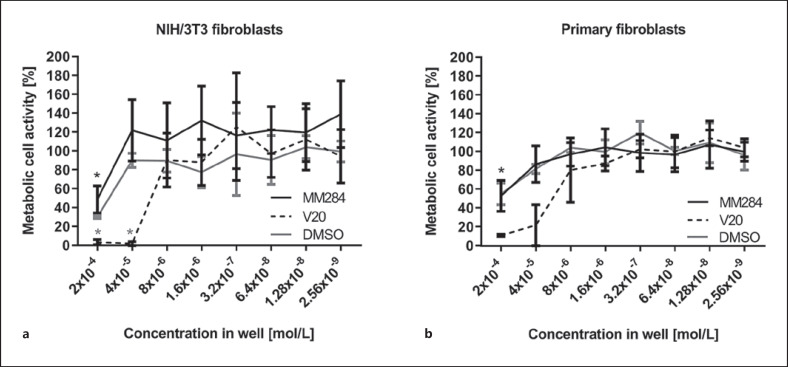
Metabolic activity of NIH/3T3 fibroblasts (*N* = 6 with *n* = 3) (**a**) and primary fibroblasts (**b**) after 48 h of incubation with immunophilin inhibitors MM284 (black line) (*N* = 6 with *n* = 3) and V20 (dashed line) (*N* = 4 with *n* = 3), and DMSO (gray line) normalized to controls (100%). Values are given as mean ± SD. *p < 0.05.

Addition of DMSO to the fibroblast cultures at a concentration of 2 × 10^−4^ mol/L resulted in a reduced viability (29% for NIH/3T3 and 53% for cochlear fibroblasts). At all lower concentrations, viability was above 75% of untreated controls and the solvent did not influence cell viability in a statistically relevant fashion (Fig. 1).

After plating of fibroblasts, treatment with V20 at concentrations of 2 × 10^−4^ mol/L and 4 × 10^−5^ mol/L resulted in no viability of NIH/3T3 fibroblasts (*p* < 0.05; Fig. 1a), whereas about 20% of cochlear fibroblasts survived at 4 × 10^−5^ mol/L (Fig. 1b). In contrast, survival of both fibroblast types was nearly unaffected at concentrations of 8 × 10^−6^ mol/L and below compared to untreated controls (*p* > 0.05 for all concentrations). After treatment with MM284, the metabolic activity of fibroblasts was not different to untreated controls for concentrations of 4 × 10^−5^ mol/L and lower for both fibroblasts (Fig. 1). At a MM284 concentration of 2 × 10^−4^ mol/L, metabolic activity was significantly (*p* < 0.05) reduced to about 50% of controls. For both types of fibroblasts, cell viability was significantly larger for MM284 compared to V20 at concentrations of 2 × 10^−4^ mol/L and 4 × 10^−5^ mol/L. Group comparison results are summarized in Table 1.

### Effects of Immunophilin Inhibitors on SGN

**Fig. 2:**
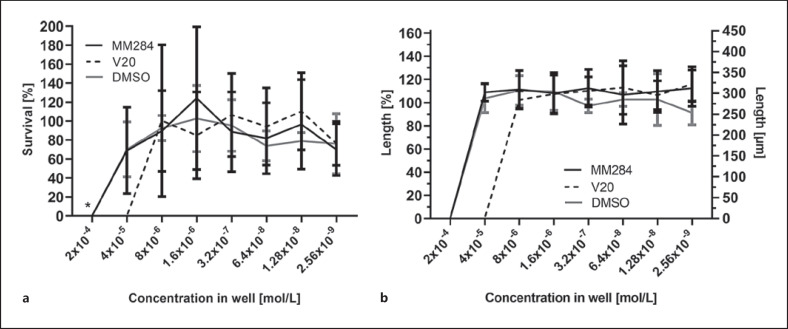
SGN survival (**a**) and neurite length (**b**) after 48 h of incubation with immunophilin inhibitors MM284 (black line) and V20 (dashed line) (*N* = 6 with *n* = 3), and DMSO (gray line) compared to the untreated controls (100%). Values are given as mean ± SD. *p < 0.05.

Incubation of SGN with different concentrations of DMSO resulted in no cell survival at a concentration of 2 × 10^−4^ mol/L, whereas at lower concentrations no statistically significant differences to untreated controls were detected (Fig. 2a). DMSO did not have an effect on neurite length of surviving SGN (Fig. 2b).

**Fig. 3:**
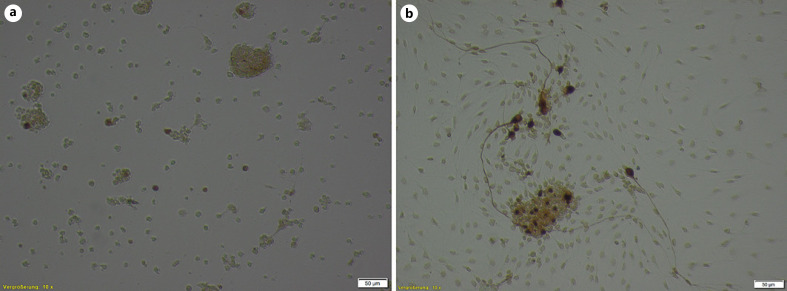
Survival and neurite growth of SGN with compound V20 (**a**) and MM284 (**b**) at a concentration of 4 × 10^−5^ mol/L after 48 h of incubation. Scale bar, 50 μm.

At concentrations of 2 × 10^−4^ mol/L, no surviving SGN were detected for both substances (Fig. 2a). The same was observed for V20 at a concentration of 4 × 10^−5^ mol/L (Fig. 3a), whereas survival of SGN increased to about 70% of controls after MM284 treatment at this concentration (Fig. 3b). For both substances and compared to controls, the survival of SGN was mostly between 80% and 110% for concentrations of 8 × 10^−6^ mol/L and below. No significant differences were detected. The highest survival rate (124%) was achieved with MM284 at 1.6 × 10^−6^ mol/L. Comparing results from different treatment groups, cell survival was significantly reduced with V20 compared to MM284 and DMSO at a concentration of 4 × 10^−5^ mol/L (Table 1).

The neurite length of the surviving SGN for BDNF-treated controls was on average 278 μm (SD: ±37 μm). When surviving cells were detected in the cultures after treatment with immunophilin inhibitors, neurite length was unaffected by the applied substances (Fig. 2b). In control and treated cultures, surviving neurons were nearly exclusively of monopolar morphology.

## Discussion

When a foreign body or implant is brought into contact with body tissue, it is exposed to a large number of immune defense reactions. This can even lead to rejection of the implant [Stolle et al., 2014]. Often involved in these processes and their regulation are CHX and cyclosporines [Flisiak and Parfieniuk-Kowerda, 2012]. In addition to the inhibition of protein synthesis by CHX, the CsA derivatives have immunomodulatory properties due to the inhibition of calcineurin via the CsA-CypA complex [Hacker and Fischer, 1993]. Their derivatives, the immunophilin inhibitors, can inhibit Cyps and are therefore potential nonimmunosuppressive candidates [Heinz­mann et al., 2015].

The current study investigated the immunophilin inhibitors MM284 and V20 with regard to a possible application in the inner ear as these should reduce the formation of connective tissue but not be toxic. The focus of this work was therefore put on their cytocompatibility for cells from the inner ear. Damage from the surgical insertion of the electrode can be categorized into immediate intracochlear changes and delayed components [Li et al., 2007; Fayad et al., 2009]. Immediate changes arise from trauma at the site of the cochleostomy or along the path of the electrode trajectory [Li et al., 2007]. Delayed changes arise from the host response to the electrode, which involves a tissue reaction consisting of inflammation, fibrosis, and possible new bone formation [Li et al., 2007; Somdas et al., 2007; Fayad et al., 2009]. As fibroblasts are a major part of the tissue formation around the CI after implantation [Somdas et al., 2007], the influence of both substances on fibroblasts (NIH/3T3 and primary cochlear fibroblasts) was investigated. The treatment of both types of fibroblasts with MM284 and V20 demonstrated unaffected metabolic cell activity at concentrations of 8*10^−6^ mol/L and below, with only V20 having affected cell viability at 4 × 10^−5^ mol/L. At 2 × 10^−4^ mol/L, reduced or nearly no cell viability was detected for both substances. This could potentially indicate toxic effects, but at a concentration of 2 × 10^−4^ mol/L also addition of DMSO without the test substances resulted in reduced cell viability. The resulting amount of DMSO at this concentration was 0.353 mol/L. It is described in the literature that DMSO shows little toxic effects at a concentration of 0.4 mol/L and is cell toxic from a concentration of 0.7 mol/L [Miller et al., 2015; Moskot et al., 2019]. This implies that at 2 × 10^−4^ mol/L, the DMSO concentration should be in a critical range, and according to our measured dose response curve, the toxic effects can most likely be attributed to the amount of DMSO in the cultures. The resulting amount of DMSO at 4 × 10^−5^ mol/L was 0.0564 mol/L. According to the literature, cell viability should nearly be unaffected at this concentration [Miller et al., 2015; Moskot et al., 2019]. This was confirmed by the presented results in the current study also for the primary cells from the inner ear. MM284 can only interact with Cyps extracellularly, which is of particular importance because the application of MM284 leads to a reduction of inflammatory processes without affecting the immune system. Using DMSO to dissolve the drug could result in the drug not acting exclusively in the extracellular space due to the increase in cell permeability [He et al., 2012]. The use of DMSO was recommended by the group of Prof. Fischer, who provided the substances. As dose response curves for DMSO and MM284 were comparable regarding cell viability and no differences between results with both drugs were detected, the metabolic cell activity of cochlear fibroblasts at a concentration of 2 × 10^−4^ mol/L appears still to be influenced by the solvent. Therefore, additional toxic effects of MM284 due to not acting exclusively extracellular are very unlikely. The higher cell viability with MM284 compared to V20 at 4 × 10^−5^ mol/L suggests that the results with V20 at this concentration are not purely caused by DMSO, but there is some toxic effect of V20. Here, it cannot be excluded that this effect is caused due to some interaction between DMSO and V20. The lack of significant differences between DMSO and V20 at 4 × 10^−5^ mol/L is probably due to the lower number of repetitions of the tests (*N* = 4). Even though it is speculation, a possible trigger for the different outcome of cochlear fibroblast at 4 × 10^−5^ mol/L could be lower doubling rates of these cells.

As any substances applied to the inner ear should not have adverse effects on SGN, both substances were also tested for their influence on survival and outgrowth of SGN. In these experiments, reduced cell survival was found at concentrations of 2 × 10^−4^ mol/L (MM284 and V20) and 4 × 10^−5^ mol/L (V20) similar to the fibroblasts with the dose response curves for DMSO alone and MM284 being undistinguishable. The toxic threshold of DMSO for hair cells is 0.176 mol/L [Qi et al., 2008], and values for SGN are not known. Therefore, we speculate that effects at 2 × 10^−4^ mol/L (DMSO concentration: 0.282 mol/L) can also be attributed to DMSO. The survival of SGN at 4 × 10^−5^ mol/L after addition of MM284 and/or DMSO indicates that the complete loss of neurons at this concentration after V20 treatment might be associated with the action of V20. The neuronal survival at concentrations of 8 × 10^−6^ mol/L and below supports the nontoxic effects of the substances at these concentrations. Neurons in SGN cultures of newborn mice can be of monopolar, bipolar, pseudomonopolar, or multipolar morphology [Whitlon et al., 2007]. As no changes in neuronal morphology were observed in surviving neurons, we speculate that MM284 has no adverse effects on the neurons in our study.

Inflammation as a factor for fibroblast proliferation often becomes apparent after insertion of a CI electrode. Among other reasons, it can be caused by mechanical tissue damage like the insertion trauma and lead to chronic inflammatory reaction, fibrosis, or new bone formation with a mostly disadvantageous growth of fibrous tissue on the implant surface [Wrzeszcz et al., 2014]. Therefore, the reduction in inflammation should also lead to a reduced formation of connective tissue around the electrode carrier. The current paper investigated the cytocompatibility of immunophilin inhibitors for a possible application in the inner ear and shall provide the basis for later in vivo experiments regarding the reduction of fibrous tissue formation after cochlear implantation.

In conclusion, MM284 can be considered as noncytotoxic for SGN and fibroblasts from the inner ear at concentrations of 4 × 10^−5^ mol/L and below and seems therefore to be a suitable candidate for an intracochlear application in vivo to investigate a possible reduction of connective tissue around the electrode carrier. At lower concentrations, the use of V20 might also be possible, but as not much information was available on this substance, we suggest gathering more information before investigating it in vivo.

## Statement of Ethics

The experiments were conducted in accordance with the German “Law on Protecting Animals” (§4) and the European Directive 2010/63/EU for protection of animals used for experimental purpose. According to the law approval for the sacrifice of animals to harvest material is not required, but it has to be registered with the local authorities (Lower Saxony State Office for Consumer Protection and Food Safety [LAVES], Oldenburg, Germany). The sacrifice of animals to harvest material was registered under no. 2016/118.

## Conflict of Interest Statement

The authors declare that they have no competing interests.

## Funding Sources

This work was supported by German Ministry for Education and Research (BMBF) as part of RESPONSE − partnership for innovation in implant technology, FKZ: 03ZZ0914D.

## Author Contributions

Madeleine Goblet: data acquisition, analysis, and writing. Thomas Lenarz: resources, supervision, interpretation, and writing. Gerrit Paasche: conceptualization, interpretation, and writing.

## Data Availability Statement

All data generated or analyzed during this study are included in this article. Further enquiries can be directed to the corresponding author.

## Figures and Tables

**Table 1:** Summary of group comparison results

Type of cell	Substance concentration	*p* value (Kruskal-Wallis test)	Comparison between		
			MM284 + V20	DMSO + V20	MM284 + DMSO
SGN	4 × 10^−5^ mol/L	0.0045	*p* < 0.01	*p* < 0.05	n.s.
NIH/3T3 fibroblasts	2 × 10^−4^ mol/L	0.0023	*p* < 0.01	n.s.	n.s.
	4 × 10^−5^ mol/L	0.0046	*p* < 0.01	n.s.	n.s.
Cochlear fibroblasts	2 × 10^−4^ mol/L	0.0198	*p* < 0.05	*p* < 0.05	n.s.
	4 × 10^−5^ mol/L	0.0208	*p* < 0.05	n.s.	n.s.

n.s., not significant. At lower concentrations, no differences were detected.
